# Reversible hearing loss after 3D video-assisted marsupialization of several posterior fossa arachnoid cysts: A case report

**DOI:** 10.1016/j.amsu.2022.103468

**Published:** 2022-03-03

**Authors:** Marouane Makhchoune, Ulysse Coneys, Michel Triffaux, Marie-Anne Labaisse, Anne Doyen

**Affiliations:** a Neurosurgery Department, Hospital Center of Wallonie Picarde, Av. Delmée 9, 7500, Tournai, Belgium; b Neurosurgery Department, Hospital Center of Wallonie Picarde, Tournai, Belgium; c Radiology Department, Hospital Center of Wallonie Picarde, Tournai, Belgium; d O.R.L Department, Hospital Center of Wallonie Picarde, Tournai, Belgium

**Keywords:** Cerebellopontine angle, Arachnoid cyst, Hypoacusis, 3D endoscopy, Case report

## Abstract

Very few pediatric cases of arachnoid cyst of ponto-cerebellar angle are described in the literature. Only 4 are described with hearing loss. It is a pathology which poses especially a problem of early diagnosis. In this paper we describe the management of a 16-year-old patient with an arachnoid cyst of the cerebellopontine angle with an isolated auditory deficit that was treated surgically. The follow up was marked by a Full recovery of hearing after surgical treatment. Arachnoid cyst of the cerebellopontine angle is rare in the pediatric population. early surgical management help to increase the chances of recovery.

## Highlights

• Very few pediatric cases of arachnoid cyst of ponto-cerebellar angle are described in the literature. Only 4 are described with hearing loss.
• This paper presents a 16-year-old patient with an arachnoid cyst of the cerebellopontine angle with an isolated auditory deficit that was treated surgically.
• The case described in this article confirms the recommendation of Giordano et al. for rapid surgical management to increase the chances of recovery.

## 1 Introduction

Arachnoid cysts are benign lesions whose internal component consists of CSF. Most often of slow and asymptomatic progression, these lesions may become symptomatic as a result of cyst enlargement or intracystic hemorrhage [[8], [9], [10], [11]]. These cysts become symptomatic during childhood in 70–90% of cases. Here we report a case of 16-year-old patient with an arachnoid cyst of the cerebellopontine angle with an isolated auditory deficit that was treated surgically.

A 16-year-old boy, right handed, without Drug, family, and psychosocial history. Was brought by his parents to the ORL consultation after recently realizing isolated left hearing loss. During a game, he realized that he couldn't hear out of his left ear and he never realized it before. So it's hard to know if it was a sudden left-sided deafness or a slowly progressive deafness. His otological history includes the installation of several *trans*-tympanic drains and the last 10-year-old audiometry was normal.

**Fig. 1:**
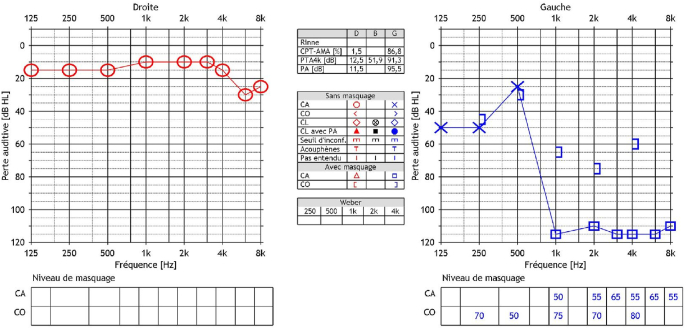
Vocal audiometrics was performed, finding a left sensorineural deafness.

**Fig. 2:**
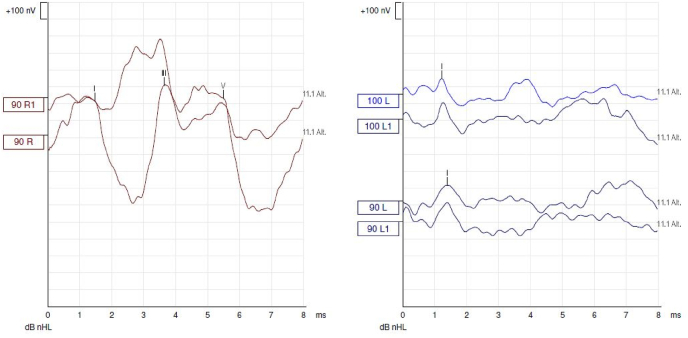
Auditory evoked potentials shows only a correctly recognizable peak 1.

An audiometric balance by tonal and vocal audiometrics was performed, finding a left sensorineural deafness of medium intensity on low frequencies and deep apart from 1 KHz, with zero understanding. Analysis of auditory evoked potentials shows only a correctly recognizable peak 1 and the examination of automated otoemissions returns to a normal response. Finally, a vestibular assessment by VHIT and video-nystagmography finds respectively normal vestibulo-ocular reflexes for the 6 semi-circular channels and a slight left vestibular deficit estimated at 22% on caloric tests with a right nystagmic preponderance (Fig. 1, Fig. 2).

Neurological examination found conscious boy without a motor or sensory deficit, the balance was normal and no nystagmus. The rest of the neurological examination was normal.

**Fig. 3:**
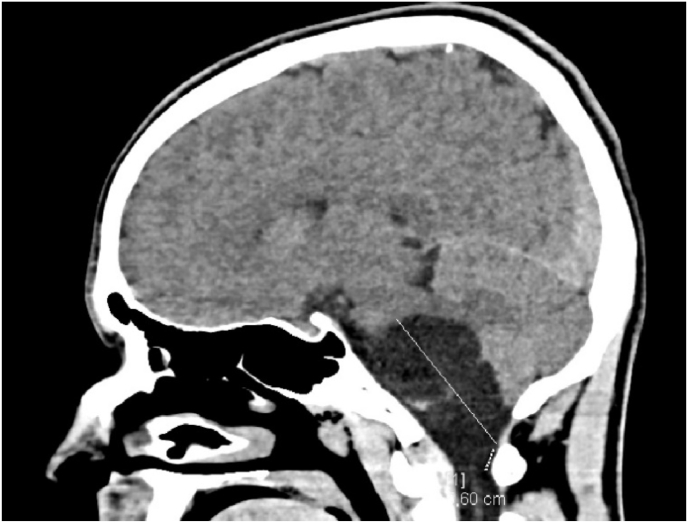
CT SCAN voluminous hypodense formation located on the left cerebellopontine angle.

A brain CT SCAN was performed revealed a voluminous hypodense formation located on the left cerebellopontine angle, causing a mass effect on the bulboprotubercular territory. This process measures 34 mm of transverse development. It is 56 mm high (Fig. 3).

**Fig. 4:**
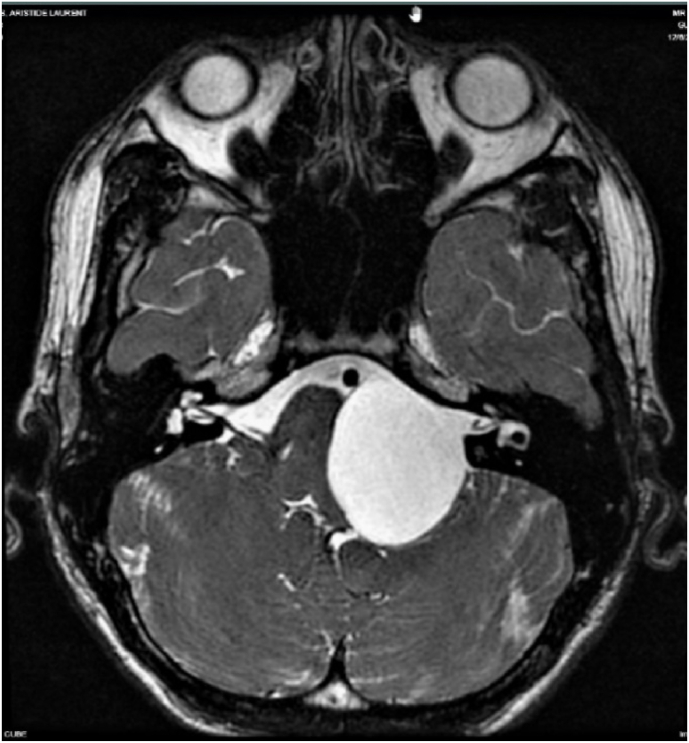
Brain mri T2 sequence showed a large cyst arachnoid compressing the brainstem and the 4th ventricle.

We thought of an arachnoid cyst and epidermoid cyst so a brain MRI was performed reveals the presence, in years the left ponto cerebelled cistern, of an extra-axial signal mass similar to the CSF in the various weights. This mass not enhanced by contrast and does not have diffusion restrictions (high CDA such as CSF), which allows the diagnosis to be directed towards a large cyst arachnoid compressing the brainstem and the 4th ventricle without hydrocephalus [14]. There is also a growing mass fet on the left acoustico-facial nervous package that is poorly visible, repressed to the posterior face of the cyst. (Fig. 4).

**Fig. 5:**
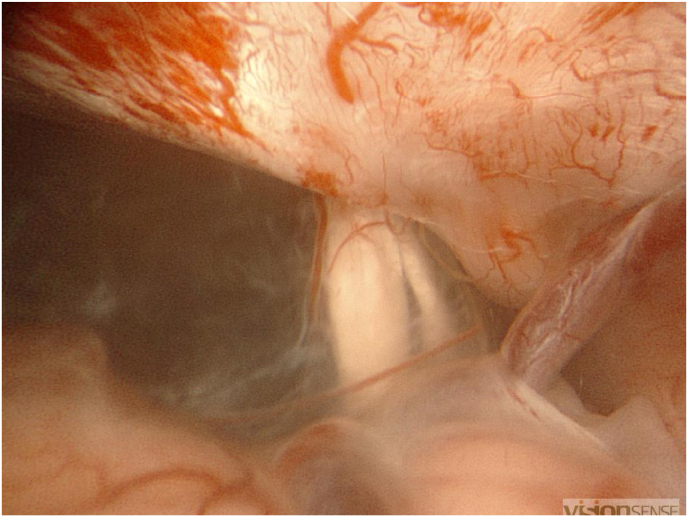
Endoscopic image of the posterior wall of the cyst.

**Fig. 6:**
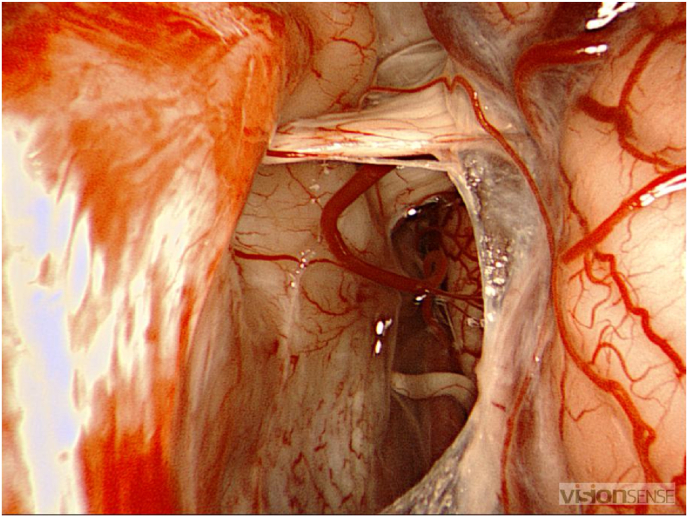
Endoscopic image showing cyst evacuation with nerve decompression.

We have retained the surgical indication The intervention was performed by our head chief department under general anesthesia. The patient was placed in a dorsal decubitus with a right tilt and rotation of the head to the right. A projection of a 2cm retro-sigmoid craniectomy respecting the sinuses. The craniectomy is performed until it reaches the limits of the sigmoid and transverse sinuses. A trident incision of the dura-mater is made. To reduce cerebellar traction, a first cyst int the left cerebellopontine cistern is opened to drain CSF. The beginning of the approach is carried out under microscope (Digital Surgical Microscope Aesculap, Inc. - a B. Braun company, Tuttlingen, Germany) until we saw the posterior wall of the arachnoid cyst. We then open it next to the acoustico-facial complex. The next steps of the surgery are performed under endoscopy 3D HD 4mm 30 (VSiii Visionsense, Philadelphia, US). A sample of the wall is taken to perform an anatomopathological analysis. After suctioning the fluid, the walls of the cysts are carefully separated from the left vertebral artery, basilar trunk, antero inferior cerebellar artery, facial nerve and vestibulocochlear nerve in order to obtain optimal ablation. Endoscopy exploration of the cystic cavity identifies and opens several other cystic walls to the trigeminal and mixed nerves. Following this dissection, good communication with the cisterns is observed (Fig. 5, Fig. 6).

No bleeding occurred during this 90-min operation. Closure of the dura-mother and filling of the craniectomy with bone powder (autograft) and tissue glue (Tisseel). No post-operative CSF leaks are found.

**Fig. 7:**
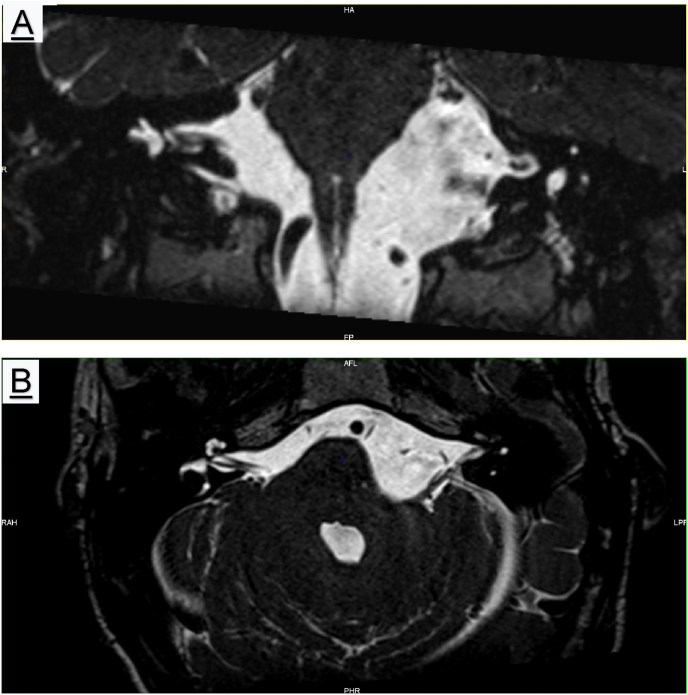
A: Postoperative MRI T2 coronal; B: Postoperative MRI T2 axial showing decompression of the nerve.

The patient spent one night in the intensive care unit before returning to the neurosurgery service. The cerebral CT-scan on the first postoperative day shows a clear reduction in the size of cystic formation of the left CPA. Impression confirmed by MRI of the fourth postoperative day which shows a good volume regression of the arachnoid cyst with a less marked mass effect on the brainstem and on the left acoustic facial nervous complex that is clearly visible on the posterior side of the cyst. Apparition of CSF flow artifacts in the cyst reflecting its communication with the cisterns of the posterior pit (Fig. 7).

**Fig. 8:**
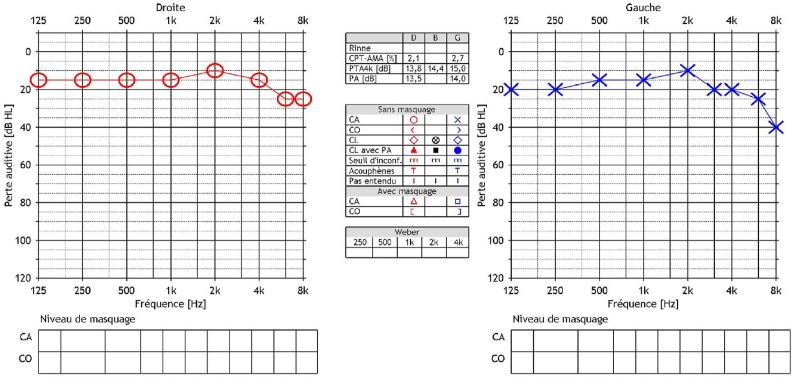
Postoperative vocal audiometrics was performed, finding complete recovery of his hearing.

The patient developed nausea and vomiting as well as mild photophobia from the second postoperative day which resolved following optimal intravenous hydration and a timely introduction of antiemetic drug (Litican®, Alizapride). No abnormalities on neurological examination are shown postoperatively, no ataxia, dysmetry or adiadococynesis are noted during the clinical examination. Audiometric tests on the fourth postoperative day show a complete recovery of his hearing (Fig. 8).

This case has been reported in line with the 2020 SCARE guidelines [17].

## 2 Discussion

As their origin is still poorly understood, different hypotheses are considered. These hypotheses include a secretion of LCR through the cyst wall and a fluid accumulation secondary to a change in osmotic gradient. Arachnoid cysts generally do not show communication with subarachnoid spaces however a third hypothesis suggests the existence of a one-way valve system allowing communication between the cyst and sub-arachnoids spaces [[9], [10], [11]].

The first reported cases of subarachnoid cysts of the cerebellopontine angle (CPA) made the subjects of a classification into 3 groups: Tumor Pseudo, Cystic and Adhesive [6,11]. With the evolution of knowledge about them, this classification has become obsolete. The first case of arachnoid cyst of the operated APC was described by Nichols and Manganiello in 1953; their patients had cerebellar disease and hearing loss. They were treated by a suboccipital approach with opening of the cyst [6].

Only 18 pediatric cases of subarachnoid cyst of APC are described in the literature. Only 4 had hearing loss. In children, they have a prevalence of 2.6% with a preponderance of boy [[1], [2], [3], [4], [5], [6],^10^]. They are most often found in the middle fossa and are in the CPA in approximately 5–6% of cases. [[1], [2], [3], [4], [5], [6], 16] In most of these cases, the initial clinical examination showed cerebellar signs associated with symptoms of intracranial hypertension.

There are only a few cases described in the literature concerning arachnoid cysts operated in a pediatric population [6]. The indication of surgery and the type of surgery is still being debated [6,8,9]. All arachnoid cysts do not require surgery and a conservative approach may be adopted in the case of an asymptomatic cyst. In these cases, a simple radio-clinical follow-up strategy is justified [6]. Surgical indication will be in cases of cyst growth, signs of compression of nearby neurovascular structures, or development of hydrocephalus [6,8]. Different surgical techniques are found in the literature; microscopic approach with cyst fenestration, marsupialization in space under arachnoid and/or resection of the cyst wall, stereotactic puncture or placement of cysto-peritoneal shunt [6,8].The microscopic approach via a retro sigmoid craniotomy is increasingly used (Table 1) and shows, combined with a resection of the cyst wall, encouraging results. However, the other techniques described in the literature present a greater risk of recurrence, and the presence of several important neurovascular structures in the CPA can make hazardous the cyst puncture or shunting [6]. The objective of each procedure being the same, put the cyst in communication with the subarachnoid spaces.

**Table 1:** Clinical review and summary of patients treated for CPA arachnoid cyst in the literature and in our case report.

Authors and Year	Age, Sex	Signs and Symptoms	Treatment, Operation	regular
Gomez et al., 1968 [13]	14 years old, F	Right spastic hemiplegia Left Spastic Hemiparesis, Left Dysmetry.	Suboccipital Craniotomy	Right hemiplegia improving in hemiparesis
Berkmen et al., 1969	5 months, H	Abnormally increased cranial perimeter, Headache	Atrio-ventricular shunt	Post-operative death.
Little et al., 1973	14 years old, F	Not described	Suboccipital Craniotomy	Not described
Sumner et al., 1975	3 years, F	Reached THE VII and VIII	Excision	Not described
Galassi et al., 1985 [12]	2 years, F	Psychomotor delay	Suboccipital Craniotomy	Normal development at 1 year.
Krisht and O'Brien, 1992	1 an, F	Vomiting and ataxia	Suboccipital Craniotomy	Vomiting recurrence and the discovery of a contralateral cyst at 2 months, the implementation of a cystoperitoneal shunt improved the patient.
Yokota et al., 1993 [15]	9 years old, F	Headache, Nystagmus Browns, Papilloedema	Cystoperitoneal shunt	Bypass review for headache
Jallo et al., 1997	14 months, H	Headache and vomiting	Retro suboccipital craniotomy-sigmoid	Asymptomatic
	3 years, H	Ataxia	Cystoperitoneal shunt	Slight improvement in ataxia but new deficit of the VII 3 weeks post-op; Resolving ataxia and deficit after recovery
	3 years, H	Dysmetry, controlateral VIII and tinnitus	Retrosigmoid suboccipital craniotomy	Dysmetry improvement; No improvement of VIII and tinnitus
	3 years, F	Headache, vomiting	Retrosigmoid suboccipital craniotomy	Asymptomatic
Boltshauser et al., 2002 [16]	5 years, ND	ND	Fenestration	ND
Ariai et al., 2005 [11]	7 years old, H	Headache, vomiting, diplopia and blurred vision	Retrosigmoid suboccipital craniotomy	Asymptomatic
Jayarao et al., 2009 [9]	12 years old, F	Hearing loss and Tinnitus	Retrosigmoid suboccipital craniotomy	Significant improvement
Olaya et al., 2011 [8]	7 years old, H	Progressive Hearing loss	Retrosigmoid suboccipital craniotomy	Full recovery
Jordan et al., 2018	14 years old, F	Headache	Retrosigmoid suboccipital craniotomy	Asymptomatic
	9 years old, F	Hearing loss	Retrosigmoid suboccipital craniotomy	Full resolution
	6 years old, H	Hearing loss	Retrosigmoid suboccipital craniotomy	Small improvement
Case described	16, H	Hearing loss	Retrosigmoid suboccipital craniotomy and cyst marsupialization	Full resolution

Hearing loss is a rare symptom, described only in 4 children of which only 2 had total recovery after surgery [[6], [7], [8]]. Its onset is thought to be the result of cochlear circulatory disorders due to the mass effect of the arachnoid cyst on the nerve complex VII-VIII. (1.6.9) A long compression period would therefore be a pejorative indicator for recovery because of vascular damage. Giordano et al. [6] suggest this because, even if it is easily conceivable that the chances of recovery are better in children because of their brain plasticity, they have noticed that a short delay between the onset of the deficit and neurosurgical treatment was associated with a better auditory result.

The use of 3D endoscopy (VSiii Visionsense, Philadelphia, US) has limited the size of craniectomy, make us able to have a better visualization of neurovascular structures such as brain stem, left vertebral artery, basilar trunk, antero-inferior cerebellar artery, trigeminal nerve, facial, vestibulocochlear and mixed nerves and thus have a good visualization of Pacchioni oval foramen at foramen magnum (Fig. 4). Its use also made possible the marsupialization of other unvisualized cysts with 3D microscopy (Digital Surgical Microscope Aesculap, Inc. - a B. Braun company, Tuttlingen, Germany). The marsupialization under 3D endoscopy (VSiii Visionsense, Philadelphia, US) is an option for the treatment of CPA arachnoids cysts which may result in a complete resolution of the patient's neurological deficit, as described in our case.

Our case supports the idea that in children, a short period of time between diagnosis and neurosurgical management is a key factor in optimal clinical improvement.

## 3 Conclusion

Cysts of the ponto-cerebellar angle are rare in the pediatric population. The delay in surgical management after the onset and/or progression of symptoms is paramount. The case described in this article confirms the recommendation of Giordano et al. for rapid surgical management to increase the chances of recovery.

## Conflicts of interest

The authors declare having no conflicts of interest for this article.

## Sources of funding

None.

## Ethical approval

Written informed consent for publication of their clinical details and/or clinical images was obtained from the patient. Ethical approval has been exempted by our institution.

## Research registration unique identifying number (UIN)

None.

## Trial registry number – ISRCTN

None.

## Author contribution

Marouane MAKHCHOUNE: Corresponding author and writing the paper

Ulysse CONEYS: writing the paper

Michel TRIFFAUX: writing the paper

Marie-Anne LABAISSE: Correcting the paper

Anne DOYEN: Correcting the paper

## Guarantor

MAKHCHOUNE MAROUANE.

## Financial disclosure

The authors declared that this study has received no financial support.

## Provenance and peer review

Not commissioned, externally peer-reviewed.
